# Supplementary material for: Association between Cholesterol Level and the Risk of Hematologic Malignancy According to Menopausal Status: A Korean Nationwide Cohort Study
Source: Biomedicines. 2022 Jul 6;10(7):1617. doi: 10.3390/biomedicines10071617 (PMC9313203; doi:10.3390/biomedicines10071617)
Supplement: Supplementary file 1 [file biomedicines-10-01617-s001.zip › biomedicines-1784964-supplementary.pdf]

biomedicines

MDPI

Supplementary Table S1. Baseline characteristics of study population.

	Total (n = 2,811,410)			
		Hematologic malignancy		
	Total	No (n=2,805,961)	Yes (n=5,449)	*p* value
Age (years)	54.0±11.5	54.0±11.5	60.2±10.8	<0.001
BMI (kg/m^2)	23.7±3.2	23.7±3.2	24.3±3.3	<0.001
WC (cm)				<0.001
<75	1,042,770(37.09)	1,041,282(37.11)	1,488(27.31)	
75-85	1,176,713(41.86)	1,174,327(41.85)	2,386(43.79)	
≥85	591,927(21.05)	590,352(21.04)	1,575(28.90)	
Total cholesterol*	200.8±42.8	200.8±42.8	199.6±38.6	0.039
HDL-C*	59.1±36.5	59.1±36.5	57.0±41.2	<0.001
LDL-C*	121.4±73.5	121.4±73.5	122.1±72.6	0.517
TG[a]*	102.51(102.45-102.58)	102.51(102.45,102.58)	113.3(111.71,114.9)	<0.001
Smoking status				<0.001
Never	2,681,898(95.4)	2,676,642(95.4)	5256(96.5)	
Ex-smoker	41,527(1.5)	41,462(1.48)	65(1.2)	
Current smoker	87,985(3.1)	87,857(3.13)	128(2.4)	
Alcohol consumption				<0.001
Non	2,252,699(80.1)	2,247,980(80.1)	4,719(86.6)	
Mild (<30 mg/d)	535,918(19.1)	535,206(19.1)	712(13.1)	
Heavy (≥30 mg/d)	22,793(0.8)	22,775(0.8)	18(0.3)	
Regular exercise	487,169(17.3)	486,216(17.3)	953(17.5)	0.753
Systolic BP**	121.8±16.1	121.8±16.1	124.8±16.1	<0.001
Diastolic BP**	75.2±10.3	75.2±10.3	76.3±10.2	<0.001
Fasting glucose*	97.0±22.1	97.0±22.1	99.5±24.4	<0.001
Hypertension, yes	908,286(32.3)	905,819(32.3)	2,467(45.3)	<0.001
Diabetes mellitus, yes	254,262(9.0)	253,510(9.0)	752(13.8)	<0.001
Dyslipidemia, yes	681,271(24.2)	679,747(24.2)	1,524(28.0)	<0.001

Data are expressed as mean ± standard deviation or number (%), except for triglycerides, which are presented as media (interquartile range) using the Wilcoxon rank-sum test. WC, Waist circumference; BP, blood pressure; eGFR, estimated glomerular filtration rate; HDL-C, high-density lipoprotein cholesterol; LDL-C, low-density lipoprotein cholesterol; TG, triglycerides. * Unit: mg/dL, ** Unit: mmHg, [a]Geometric mean (95% confidence interval).

Supplementary Table S2. Unadjusted and adjusted risks of hematologic malignancy by quartile of each lipid profile according to menopausal status.

Lipid profile			Premenopause (n = 1,189,806)			Postmenopause (n = 1,621,604)		
			Model 1 (Crude) HR (95% CI)	Model 2 aHR (95% CI)	Model 3 aHR (95% CI)	Model 1 (Crude) HR (95% CI)	Model 2 aHR (95% CI)	Model 3 aHR (95% CI)
TC	Q1	<175	1(Ref.)	1(Ref.)	1(Ref.)	1(Ref.)	1(Ref.)	1(Ref.)
	Q2	175-197	0.92(0.79,1.07)	0.87(0.75,1.01)	0.85(0.73,0.99)	0.80(0.73,0.87)	0.81(0.74,0.89)	0.81(0.74,0.88)
	Q3	198-223	1.07(0.92,1.25)	0.96(0.83,1.12)	0.93(0.80,1.08)	0.74(0.68,0.80)	0.76(0.70,0.83)	0.75(0.69,0.82)
	Q4	≥224	1.08(0.92,1.29)	0.90(0.76,1.07)	0.86(0.73,1.03)	0.66(0.60,0.71)	0.68(0.62,0.74)	0.67(0.61,0.73)
HDL-C	Q1	<48	1(Ref.)	1(Ref.)	1(Ref.)	1(Ref.)	1(Ref.)	1(Ref.)
	Q2	48-55	0.88(0.74,1.03)	0.90(0.76,1.06)	0.91(0.77,1.08)	0.80(0.74,0.87)	0.84(0.78,0.91)	0.85(0.79,0.92)
	Q3	56-65	0.89(0.76,1.04)	0.92(0.79,1.08)	0.95(0.81,1.11)	0.77(0.71,0.83)	0.83(0.76,0.90)	0.84(0.78,0.91)
	Q4	≥66	0.73(0.62,0.86)	0.76(0.64,0.90)	0.80(0.68,0.95)	0.62(0.56,0.67)	0.68(0.62,0.75)	0.70(0.64,0.77)
LDL-C	Q1	<96	1(Ref.)	1(Ref.)	1(Ref.)	1(Ref.)	1(Ref.)	1(Ref.)
	Q2	96-116	0.99(0.85,1.15)	0.94(0.81,1.10)	0.92(0.79,1.07)	0.86(0.79,0.94)	0.88(0.81,0.97)	0.88(0.80,0.96)
	Q3	117-140	1.10(0.94,1.28)	1.00(0.85,1.16)	0.96(0.82,1.12)	0.84(0.77,0.91)	0.87(0.80,0.94)	0.86(0.79,0.94)
	Q4	≥141	1.12(0.94,1.32)	0.95(0.80,1.13)	0.90(0.75,1.07)	0.72(0.66,0.78)	0.75(0.69,0.81)	0.74(0.68,0.80)
TG	Q1	<72	1(Ref.)	1(Ref.)	1(Ref.)	1(Ref.)	1(Ref.)	1(Ref.)

Q2	72-100	1.18(1.02,1.37)	1.11(0.96,1.29)	1.09(0.94,1.27)	1.09(0.98,1.21)	1.01(0.91,1.12)	1.00(0.90,1.11)
Q3	101-144	1.15(0.98,1.35)	1.03(0.88,1.22)	0.99(0.84,1.17)	1.22(1.11,1.35)	1.08(0.98,1.19)	1.05(0.96,1.16)
Q4	≥145	1.51(1.29,1.78)	1.30(1.10,1.53)	1.22(1.02,1.44)	1.19(1.09,1.31)	1.02(0.93,1.12)	0.98(0.89,1.08)

Model 2 was adjusted for age. Model 3 was adjusted for age, body mass index, smoking, alcohol consumption, regular exercise, diabetes mellitus, and history of taking medication for dyslipidemia within a year. TC, total cholesterol; HDL-C, high-density lipoprotein cholesterol; LDL-C, low-density lipoprotein cholesterol; IR, incidence rate; PY, person years; aHR, adjusted hazard ratio; CI, confidence interval; TG, triglycerides.

Supplementary Table S3. Incidence rates of subtypes of hematologic malignancy by quartile of each lipid profile.

		Premenopause (n = 1,189,806)			Postmenopause (n = 1,621,604)			Premenopause (n = 1,189,806)			Postmenopause (n = 1,621,604)		
		Subjects (N)	Event (n)	IR (per 10^6 PYs)	Subjects (N)	Event (n)	IR (per 10^6 PYs)	Subjects (N)	Event (n)	IR (per 10^6 PYs)	Subjects (N)	Event (n)	IR (per 10^6 PYs)
		**Multiple myeloma**						**Non-hodgkin's lymphoma**					
TC	Q1	399,781	82	24.7	310,748	368	144.8	399,781	143	43.0	310,748	355	139.7
	Q2	332,944	67	24.2	360,025	314	105.8	332,944	122	44.1	360,025	344	116.0
	Q3	269,814	52	23.2	430,213	348	98.0	269,814	103	45.9	430,213	411	115.7
	Q4	187,267	35	22.5	520,618	388	90.3	187,267	85	54.6	520,618	402	93.5
HDL-C	Q1	241,750	64	31.8	470,378	538	139.4	241,750	103	51.1	470,378	507	131.4
	Q2	277,350	48	20.8	402,498	363	109.4	277,350	120	52.0	402,498	368	110.9
	Q3	333,451	70	25.3	400,215	315	95.4	333,451	124	44.8	400,215	367	111.1
	Q4	337,255	54	19.3	348,513	202	70.2	337,255	106	37.9	348,513	270	93.9
LDL-C	Q1	373,625	77	24.8	327,897	344	128.3	373,625	133	42.8	327,897	361	134.6
	Q2	337,040	60	21.4	352,114	301	103.8	337,040	118	42.1	352,114	351	121.1
	Q3	284,577	65	27.5	427,630	381	107.9	284,577	112	47.3	427,630	396	112.2
	Q4	194,564	34	21.0	513,963	392	92.3	194,564	90	55.6	513,963	404	95.1
TG	Q1	438,153	75	20.6	275,406	210	92.1	438,153	143	39.3	275,406	224	98.2
	Q2	322,417	71	26.5	364,952	309	102.6	322,417	134	50.0	364,952	315	104.6
	Q3	247,407	43	20.9	456,537	414	110.2	247,407	94	45.7	456,537	474	126.2
	Q4	181,829	47	31.1	524,709	485	112.5	181,829	82	54.3	524,709	499	115.8
		**Hodgkin's lymphoma**						**Lymphoid leukemia**					
TC	Q1	399,781	10	3.0	310,748	43	16.9	399,781	45	13.5	310,748	74	29.1
	Q2	332,944	9	3.3	360,025	29	9.8	332,944	33	11.9	360,025	81	27.3
	Q3	269,814	16	7.1	430,213	40	11.3	269,814	22	9.8	430,213	79	22.2
	Q4	187,267	6	3.9	520,618	36	8.4	187,267	18	11.6	520,618	85	19.8
HDL-C	Q1	241,750	7	3.5	470,378	52	13.5	241,750	27	13.4	470,378	111	28.8
	Q2	277,350	8	3.5	402,498	43	13.0	277,350	34	14.7	402,498	75	22.6
	Q3	333,451	13	4.7	400,215	38	11.5	333,451	29	10.5	400,215	77	23.3
	Q4	337,255	13	4.6	348,513	15	5.2	337,255	28	10.0	348,513	56	19.5
LDL-C	Q1	373,625	11	3.5	327,897	42	15.7	373,625	33	10.6	327,897	79	29.5
	Q2	337,040	12	4.3	352,114	31	10.7	337,040	41	14.6	352,114	70	24.1
	Q3	284,577	13	5.5	427,630	34	9.6	284,577	26	11.0	427,630	85	24.1
	Q4	194,564	5	3.1	513,963	41	9.7	194,564	18	11.1	513,963	85	20.0
TG	Q1	438,153	15	4.1	275,406	21	9.2	438,153	40	11.0	275,406	41	18.0
	Q2	322,417	9	3.4	364,952	32	10.6	322,417	32	11.9	364,952	82	27.2
	Q3	247,407	9	4.4	456,537	40	10.6	247,407	24	11.7	456,537	99	26.3
	Q4	181,829	8	5.3	524,709	55	12.8	181,829	22	14.6	524,709	97	22.5
		**Myeloid leukemia**											
TC	Q1	399,781	141	42.4	310,748	265	104.3						
	Q2	332,944	98	35.4	360,025	253	85.3						
	Q3	269,814	106	47.2	430,213	264	74.3						
	Q4	187,267	70	45.0	520,618	304	70.7						
HDL-C	Q1	241,750	97	48.2	470,378	376	97.4						
	Q2	277,350	97	42.0	402,498	257	77.4						
	Q3	333,451	124	44.8	400,215	255	77.2						
	Q4	337,255	97	34.7	348,513	198	68.8						

LDL-C	Q1	373,625	124	39.9	327,897	261	97.3
	Q2	337,040	112	40.0	352,114	247	85.2
	Q3	284,577	106	44.8	427,630	276	78.2
	Q4	194,564	73	45.1	513,963	302	71.1
TG	Q1	438,153	130	35.7	275,406	177	77.6
	Q2	322,417	113	42.2	364,952	237	78.7
	Q3	247,407	82	39.9	456,537	308	82.0
	Q4	181,829	90	59.5	524,709	364	84.4

TC, total cholesterol; HDL-C, high-density lipoprotein cholesterol; LDL-C, low-density lipoprotein cholesterol; TG, triglycerides; IR incidence rate, PY person years.
